# P20 - The use of regulatory hexapeptide on frequently ill children

**DOI:** 10.1186/2045-7022-4-S1-P75

**Published:** 2014-02-28

**Authors:** Maleyka Karimova

**Affiliations:** 1 Azerbaijan Medical University, Baku, Azerbaijan

## 

The use of regulatory hexapeptide at frequently ill children Background. Respiratory diseases is one of the actual problems of modern pediatrics. The aim is to study clinical and laboratory level performance imunofan ( regulatory hexapepdide- alpha-arginyl-lysyl-aspartyl-valyl-tyrosyl-arginine) at frequently ill children (FIC). Material and methods.To this end, 42 children were examined frequently ill with acute respiratory disease. The levels of cytokines IL-1beta, IL-2, IL-6, IL-8, TNF-alpha, IFN-gamma, serum level of substance P, condition hemostasis ,of CD3, CD4, CD8, CD19 cell, content immunoglobulins A, M, G, E. It was also studied the intestinal flora, microbiocenosis nasopharynx. Results.It was found that in the acute period FIC marked increase in levels of IL-1beta, IL-6,IL-8, TNF-alpha and substance P, blood hypercoagulation, reduced IL-2 and IFN -gamma. Clinical remission was not accompanied by normalization of indicators. The high level of pro-inflammatory cytokines, substance P and IgM, a tendency to hypercoagulability in the period of clinical remission in FIC, reflects ongoing inflammatory process, which in our opinion, due to the persistence of the infectious agent. A significant reduction in IL-2, IFN-gamma these children, due to the presence of secondary immunodeficiency cell type. This is evidenced by the reduced number of cellular immunity (CD3, CD4, and the index of CD4 / CD8). In conclusion. Application regulatory hexapepdide- imunofan at FIC leads to positive dynamics of immunological parameters, sanitizes lasting infections, eliminates bacteria overgrowth, prevent complications and relapses. Imunofan has established itself as a drug having anti-inflammatory activity in the treatment of respiratory opportunistic infections.

